# Immune Defect in Adults With Down Syndrome: Insights Into a Complex Issue

**DOI:** 10.3389/fimmu.2020.00840

**Published:** 2020-05-08

**Authors:** Yannick Dieudonné, Beatrice Uring-Lambert, Mohamed Maxime Jeljeli, Vincent Gies, Yves Alembik, Anne-Sophie Korganow, Aurélien Guffroy

**Affiliations:** ^1^INSERM UMR – S1109, Université de Strasbourg, Strasbourg, France; ^2^Department of Clinical Immunology and Internal Medicine, National Reference Center for Systemic Autoimmune Diseases (CNR RESO), Tertiary Center for Primary Immunodeficiency, Hôpitaux Universitaires de Strasbourg, Strasbourg, France; ^3^Faculty of Medicine, Université de Strasbourg, Strasbourg, France; ^4^Department of Immunobiology, Hôpitaux Universitaires de Strasbourg, Strasbourg, France; ^5^INSERM U1016, Département 3I Infection, Immunité et Inflammation, Institut Cochin, Université de Paris, Paris, France; ^6^Department of Immunobiology, Hôpital Cochin, AP-HP-Centre Université de Paris, Paris, France; ^7^Faculty of Pharmacy, Université de Strasbourg, Illkirch, France; ^8^Department of Clinical Genetic, Hôpitaux Universitaires de Strasbourg, Strasbourg, France

**Keywords:** down syndrome, adulthood, primary immunodeficiency, autoimmunity, infectious risk

## Abstract

Children with Down syndrome (DS) suffer from recurrent respiratory infections, which represent the leading cause of mortality during childhood. This susceptibility to infections is usually considered multifactorial and related to both impaired immune function and non-immunological factors. Infections are also one of the top causes of death in DS at adulthood. DS is considered an immunodeficiency with syndromic features by some researchers because of this high rate of infection and the immunological characteristics observed in children with DS. Little is known about the immune status of adult patients. Herein, we report the clinical and immune phenotype of 44 adults with DS, correlated with their infectious history. We observed that these adults had an aberrant lymphocyte phenotype with decreased naïve/memory T cell ratios and reduced numbers of switched memory B cells. The lower incidence of infectious events at adulthood distinguish DS from other inborn errors of immunity. Primary immunodeficiency-related features in DS could explain the increased risk of developing autoimmunity, malignancies, and infections. During adulthood, this immune dysfunction may be compensated for in mid-life, and infection-related mortality observed in older patients might be favored by multiple factors such as neurological impairment or nosocomial antigen exposure.

**Clinical Trial Registration:** www.ClinicalTrials.gov, identifier NCT01663675 (August 13, 2012).

## Introduction

**FIGURE 1:**
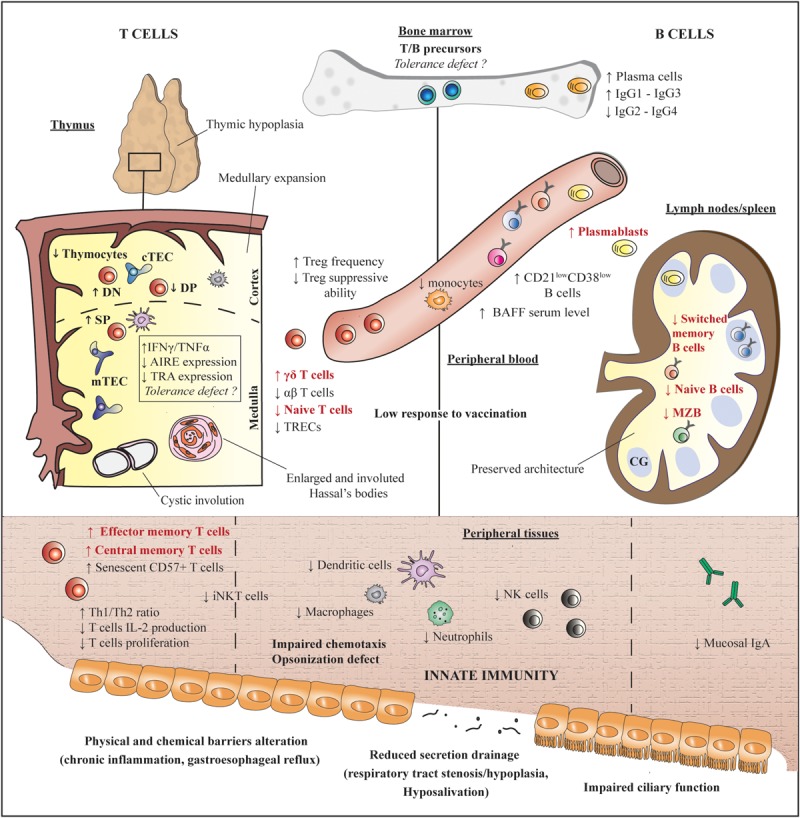
Immunological abnormalities in children with DS. Features found in our adult patients are depicted in red. References are detailed in Supplementary Table 1. AIRE, autoimmune regulator; BAFF, B cell activating factor; cTEC, cortical thymic epithelial cells; DN, double negative; MZB, marginal zone-like B cells; mTEC, medullary thymic epithelial cells; NK, natural killer; SP, simple positive; TRA, tissue restricted antigen; TREC, T cell receptor excision circle.

Down syndrome, resulting from a partial or complete triplication of chromosome 21, is the most frequent viable chromosome abnormality in humans. It is associated with various health issues, including intellectual disability, hypotonia, and congenital malformations, with a high incidence of cardiac anomalies (1, 2). Children also suffer from recurrent respiratory infections, which represent the leading cause of mortality during this period. This susceptibility to infections is usually considered to be multifactorial and related to both immunological disorders and other factors like abnormal airway anatomy or possible cilia dysfunction (3–5). Additionally, patients also have a high frequency of hematological malignancies and autoimmune diseases, such as hypothyroidism or celiac disease (6). Several immune defects are reported in children with DS (Figure 1 and Supplementary Table 1), and some authors hence argued to classify DS as an inborn error of immunity. Inadequate vaccinal responses and decreased IgG2 or IgG4 immunoglobulin levels are suggestive of a primary antibody deficiency. Proportions of B cell populations resemble CVID patterns, with a decrease of switched memory B cells and an increased number of likely autoreactive CD21^low^ B cells (7, 8). The T cell compartment is also compromised by abnormal thymic architecture, a defect of thymocyte and naïve T cell development, and an expansion of memory T cells (9). Finally, studies focusing on innate immunity reported an increased frequency of NK cells with reduced suppressive function and defective neutrophils chemotaxis (Figure 1 and Supplementary Table 1) (3, 6).

Due to medical advances and improved surgical treatments for congenital heart diseases, life expectancy of DS patients has considerably increased over the past decades, so that it now exceeds 60 years. Health conditions of adult patients, especially regarding immunity, are not well known, but become an important issue when considering that respiratory infections represent the primary cause of death in DS (2).

To determine how immunological abnormalities evolve at adulthood, we collected medical histories, including retrospective prevalence of infections, autoimmune manifestations, and malignancies, as well as serological and immunobiological parameters (at one point) from adult patients with DS.

## Materials and Methods

### Study Approval

This study was conducted in accordance with the principles of the Helsinki declaration, and approved by the institutional Ethical Board at Strasbourg University Hospital (CPP-Est IV N°12/47). All participants (or their parents) provided written consent for enrollment in this study.

### Patients

Patients were recruited in a study of social and medical conditions of adult patients with DS in Alsace (France, Strasbourg University Hospital, PHRC 2012-A00466-37). All consecutive patients over 18 years of age and a cytogenetic diagnosis of trisomy 21, cared for by the genetical department of our tertiary center between 2014 and 2018, were proposed to participate and were enrolled by board-certified clinical geneticists. All general practitioners from Alsace and a specific patient association (ADAPEI) were contacted. Patients who agreed to participate were referred to the genetical department for enrollment. Pregnant patients and those who were unable to travel due to severe comorbid conditions were excluded.

### Clinical Data Collection

For all patients medical history was collected using a standardized questionnaire filled by the patient and a family member or legal representative with the help of a clinician. These declarative data were systematically completed by examination of medical records (including retrospective assessment of childhood events noted in the patients’ health booklets). Notably, occurrences of congenital cardiac malformations, autoimmune diseases, infections, malignant diseases, and vaccinations (type and date) were systematically recorded. Recurrent infections were defined as infections requiring at least two courses of oral antibiotics over a 12-month period.

### Clinical Laboratory Evaluations

Laboratory evaluations were performed in the Laboratory of Immunology (Strasbourg University Hospital).

Sera submitted for anti-nuclear antibodies were analyzed by immunofluorescence using HEp-2 cells (HEp-2 cell slides and anti-human IgG, IgA, and IgM fluorescent conjugates were obtained from ZEUS Scientific, Raritan, NJ, United States), and titrated when positive (≥1/320). Presence of anti-thyroperoxydase (TPO) antibodies and anti-transglutaminase antibodies was assessed by fluorescence enzyme immunoassays on a Phadia^®^ instrument (Thermo Fisher scientific Inc, Uppsala, Sweden). Immunoglobulin levels and post-vaccination serology (tetanus IgG kit, The Binding Site Group Ltd, Birmingham, United Kingdom for tetanus; IgG ELISA according to WHO guidelines for *Streptococcus pneumoniae)* were also checked. Antibodies titers >0.15 IU/mL were considered protective against tetanus (10). Immune protection against *Streptococcus pneumoniae* was defined by an antigen-specific IgG concentration ≥1.3 μg/mL for at least 70% of tested serotypes (1, 3, 4, 5, 6A, 6B, 7F, 9V, 10A, 12F, 14, 15B, 18C, 19A, 19F, and 23F) (11, 12). Lymphocyte phenotype analyses were performed using routinely validated combinations of monoclonal antibody panels on a Navios^®^ flow cytometer (Beckman-Coulter Inc, California, United States). The distribution of B cells subset was compared to that of 27 age-matched healthy controls.

### Statistical Analysis

Data are presented as median (range) or frequency (percentage). Statistical significance was calculated with two-tailed unpaired Mann-Whitney *U*-test. *P* values lower than 0.05 were considered as statistically significant. Data were analyzed using GraphPad Prism software version 7 (GraphPad software Inc, San Diego, CA, United States).

## Results

Among 51 identified patients, seven were excluded: two who declined to take part in the study and five with severe comorbid conditions. Forty-four adult patients (>18 years) with a confirmed diagnosis of regular trisomy 21 (i.e., all patients had a complete third copy of chromosome 21, and none of them had translocation or mosaicism) were included, with a median age of 31 years (Table 1). Twenty-eight patients (64%) lived with a family member, 18 (42%) in a specialized institution. Congenital heart diseases were found in 13 patients (30%), macroglossia in 11 (25%), with no pulmonary airway malformations. Two patients suffered from epilepsy, none from dementia. In accordance with other studies, 23 (52%) experienced recurrent infections during childhood, mostly lower respiratory tract (*n* = 20, 45%) and ear-nose-throat (ENT, *n* = 7, 13%) infections. One patient exhibited extensive varicella-zoster virus (VZV) infection with throat involvement. His clinical condition improved within a few days without sequelae, and he did not experience viral reactivation during the following years.

**TABLE 1:** Clinical and immunological features of adult patients with DS.

	**Reference values**	**Patients (*n* = 44)**
Age (years)		31 (18–52)
Sex ratio (F/M)		1 (22/22)
Clinical features (*n*, %)		
Recurrent infections		23 (53%)
Before 18 years		
Respiratory infections		20 (45%)
ENT infections		7 (13%)
Opportunistic infections		1 (2%)*
After 18 years		
Respiratory infections		1 (2%)
ENT infections		1 (2%)
Opportunistic infections		0
Autoimmune manifestations		23 (52%)
Hypothyroidy		23 (52%)
Celiac disease		2 (5%)
Malignant disease		0
Biology		
ANA positivity (*n*, %)	<1/320	7 (16%)
Anti-DNA antibodies (*n*, %)	<50 U/ml	2 (5%)
Anti-TPO antibodies (*n*, %)	<34 kU/l	4 (9%)
IgG (g/L)	7.2–14.7	14.3 (10.5–21.2)
IgA (g/L)	1.1–3.6	3.1 (1.6–7.3)
IgM (g/L)	0.5–3.1	0.8 (0.3–2.9)
T cells (cells/μL)	700–1,900	1,167 (515–2,516)
CD4^+^ T cells (cells/μl)	400–1,300	586 (273–1,307)
Naïve CD4^+^ T cells (% CD4^+^)	26–54	25 (13–35)
Central memory CD4^+^ T cells (% CD4^+^)	28–51	36 (26–42)
Effector memory CD4^+^ T cells (% CD4^+^)	8–23	30 (19–39)
CD8^+^ T cells (cells/μl)	200–700	449 (102–1,365)
Naïve CD8^+^ T cells (% CD8^+^)	24–53	14 (4–21)
Central memory CD8^+^ T cells (% CD8^+^)	5–19	5 (1–7)
Effector memory CD8^+^ T cells (% CD8^+^)	16–33	44 (24–51)
TEMRA CD8^+^ T cells (% CD8^+^)	5–37	33 (19–50)
Treg cells (% CD4^+^)	2.7–5	2.6 (1.4–4.3)
γδ T cells (% CD3^+^)	2–6	8 (1–12)
B cells (cells/μL)	169–271	72 (37–263)
Naive B cells (cells/μL)	112–169	49 (4–247)
Transitional B cells (cells/μL)	2–6	2 (1–8)
Switched memory B cells (cells/μL)	18–40	9 (2–22)
Marginal zone-like B cells (cells/μL)	22–54	2 (1–16)
Plasmablasts (cells/μL)	1–3	3 (0–13)
CD21^low^CD38^low^ (cells/μL)	4–11	2 (1–11)
NK (cells/μl) (cells/μL)	100–400	223 (54–633)

*Extensive varicella-zoster virus primo-infection. ANA, antinuclear antibodies; ENT: ear-throat-nose; F, female; M, male; NK, natural killer. Frequency (percentage) and median (range).

Only one patient had recurrent infections beyond age 18, manifesting in repeated bronchitis. He did not suffer from congenital cardiac malformation or cardiopulmonary comorbidities. Twenty-three (52%) patients had a history of autoimmune diseases, including hypothyroidism needing hormonal replacement therapy (*n* = 23), and symptomatic celiac disease positive for anti-transglutaminase antibodies (*n* = 2). Anti-nuclear antibodies were found in seven patients (16%); with anti-DNA specificity in two patients but without any signs of systemic auto-immune disease. Nineteen (43%) had hypergammaglobulinemia involving IgG (>15g/L).

**FIGURE 2:**
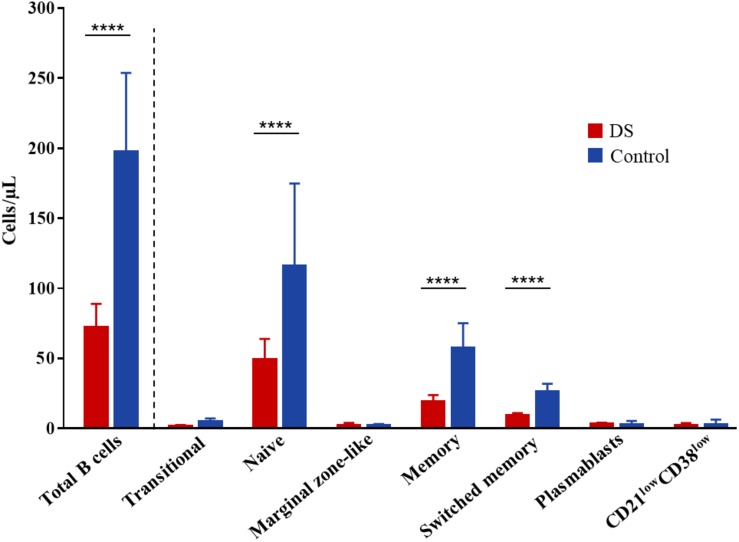
Absolute numbers of B cell subsets in adult patients with DS and age-matched control subjects. ****P < 0.0001. DS, Down syndrome.

Circulating lymphocyte subpopulations were evaluated for all patients (see Supplementary Figure 1 for the gating strategy). Most of them had moderate lymphopenia (*n* = 35, 80%). Despite normal T cell numbers, patients had a reduced percentage of naïve T cells, and an increased frequency of effector memory T cells in both CD4^+^ and CD8^+^ compartments (Table 1). Percentages of regulatory T (Treg) cells were within the normal range. Notably, patients presented low CD19^+^ B cell blood counts, and, when compared with age-matched controls, a reduced number of switched memory (9 vs. 26 cells/μL, p < 0.0001) and naïve (49 vs. 116 cells/μL, p < 0.0001) B cells was observed, with similar counts of plasmablasts and CD21^low^ cells (Figure 2). Regarding the immunoglobulin levels, almost all patients had normal levels of IgA, IgM, and IgG, and normal IgG subclass distribution. Low serum IgM levels were noted in two patients, and isolated low levels of IgG4 in two others. The patient with recurrent infections in adulthood showed increased IgA and normal IgG and IgM levels, but presented with mild lymphopenia (1,300/mm^3^) with predominant B cell lymphopenia (53/mm^3^), and a reduced frequency of switched memory B cells (13% of CD19 + cells).

**FIGURE 3:**
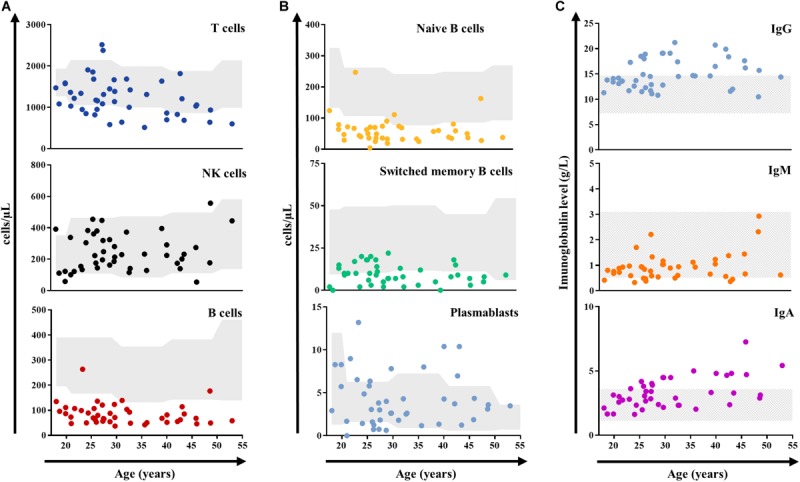
Absolute count (cells/μl) of peripheral blood lymphocytes **(A)**, B-cell subsets **(B)**, and immunoglobulin levels (g/L, **C**) distributed by age in adult patients with DS (*n* = 44). Gray shading represents the age-matched normal range (10–90th percentile) from a French cohort for lymphocytes (13), and reference laboratory values for immunoglobulins. DS, Down syndrome; NK, natural killer.

Among the 37 patients tested, 36 (92%) had protective antibody titers against tetanus. Three patients were vaccinated against S*treptococcus pneumoniae*. According to post-vaccination testing, none of them had a protective immune status but all mounted a partial response (seroconversion was observed for, respectively, 8, 9, and 11 serotypes, Supplementary Table 2). The age-related distribution of T cells and immunoglobulins resembled that from healthy donors (13, 14), with a progressively decrease of T cells and increase of serum immunoglobulin levels, whereas B cell numbers remained low across the adult life (Figure 3).

## Discussion

Down syndrome has a heterogeneous pattern of clinical manifestations. Some are frequent, such as cardiac malformations or hypothyroidism, while others have frequencies or expression patterns that are more variable, like hematopoietic malignancies or infectious events. More specifically, mechanisms underlying immune disorders in DS remain poorly understood and have alternatively been interpreted as a precocious aging or an intrinsic defect of the immune system (6). Different genes mapping to chromosome 21 are involved in immune function (*ITG*β*2*, *IFNAR1*, *IFNGR2*, and *ICOSL*, etc.), but their overexpression through gene dosage effects has not been proven (9). Several epigenetic-related genes are also encoded on this chromosome, which might rather alter expression of key actors of B cell or T cell development (i.e., *AIRE*) (15).

Despite persistent immune abnormalities, and especially a reduced number of switched memory B cells (Figure 1), most of our patients do not display susceptibility to infection at adulthood. The low frequency of opportunistic infections is in accordance with a previous work from Schoch et al. showing preserved VZV- and CMV- specific immunity in children and adults with DS (16). The reduced frequency of infections at adulthood compared to childhood distinguish DS from other primary immune deficiencies and is probably multifactorial. It might be related to the polygenic character of the disease, and the epigenetic modulation of the multiple genes expression due to chromosome 21 triplication in a given patient (9, 15). An alternative or complementary hypothesis is that the immune system may mature with age in DS.

Kuster et al. indeed suggested that repeated antigen contact could induce an adequate maturation level for the humoral immune system, which could explain why infections subside after 6–8 years of age (17). In our series, most of the patients had immune protection against tetanus. Only three patients were vaccinated against *Streptococcus pneumoniae*. The one recently vaccinated mounted a protective response, which was found to be incomplete in the two patients with longer delay since vaccination. Although this post-vaccination response evaluation is limited, these results, combined to the absence of hypogammaglobulinemia, suggest that adult DS patients do not have a complete humoral defect. Besides, a similar improvement with age has been reported in children with impairment of TLR signaling (IRAK-4 and Myd88 deficiencies), in whom bacterial infections become rarer with age (18). Some authors argued that other innate pathways may progressively play a compensatory role (19). Similarly, innate immune system may also mature to prevent further infections in older children and adults with DS. Further studies including response to combined pneumococcal vaccine (13 and 23 valences) assessment and functional evaluation of innate immune actors, are needed to elucidate these issues in adults with DS.

Our results might be surprising considering studies that identify respiratory infections as the first cause of mortality in DS (2, 20). This might be partly explained by the relatively young age of the patients, the enrollment method limited to consultations, and the low incidence of neurological diseases in our cohort. Indeed, our results must be put in perspective with studies that identify a high incidence of age-related comorbid conditions in adult with DS, including an exceptional risk for developing Alzheimer’s disease, especially after 50 years of age (21, 22). We previously showed that respiratory infections were associated with dementia and late onset epilepsy in hospitalized DS patients, especially after age 40 (23). These key markers, in addition to extrinsic factors such as changes in antigen exposure or lifestyle with age, might be a possible explanation for the reoccurrence of infections over time.

## Conclusion

In conclusion, susceptibility to infections, which is sustained by the persistence of immunological abnormalities described in children, may remain clinically quiescent in young adult with DS until additional factors come into play, such as neurological manifestations or nosocomial antigen exposure. Despite our limited post-vaccination response evaluation, the present work underlines the need of more comprehensive studies evaluating strategies to improve care of adult patients with DS, ideally combining a careful screening for neurological comorbidities and a dedicated vaccination program.

## Data Availability Statement

All datasets generated for this study are included in the article/Supplementary Material.

## Ethics Statement

The studies involving human participants were reviewed and approved by the Institutional Ethical Board at Strasbourg University Hospital (CPP-Est IV N°12/47). The patients/participants provided their written informed consent to participate in this study.

## Author Contributions

AG, YA, A-SK, and YD contributed to conception and design of the study. AG, YA, BU-L, and MJ enrolled the patients and performed the experiments. YD, AG, YA, A-SK, BU-L, VG, and MJ analyzed the data and organized the database. YD, AG, VG, and A-SK wrote the manuscript. All authors contributed to manuscript revision, read, and approved the submitted version.

## Conflict of Interest

The authors declare that the research was conducted in the absence of any commercial or financial relationships that could be construed as a potential conflict of interest.

## Abbreviations

AIRE: autoimmune regulator
BAFF: B cell activating factor
CMV: cytomegalovirus
cTEC: cortical thymic epithelial cell
CVID: common variable immunodeficiency
DS: down syndrome
ENT: ear nose and throat
HEp-2: human epithelial type 2
IFN: interferon
iNKT: invariant natural killer T cell
mTEC: medullary thymic epithelial cell
MZB: Marginal zone-like B cell
NK: natural killer
TEMRA: T effector memory RA^+^
TPO: thyroperoxidase
TRA: tissue restricted antigen
TREC: T cell receptor excision circle
Treg: T regulatory cells
VZV: varicella-zona virus.
