# Supplementary material for: Immune Defect in Adults With Down Syndrome: Insights Into a Complex Issue
Source: Front Immunol. 2020 May 8;11:840. doi: 10.3389/fimmu.2020.00840 (PMC7225335; doi:10.3389/fimmu.2020.00840)
Supplement: Supplementary file 1 [file Data_Sheet_1.docx]

**Immune defect in adults with Down syndrome: insights into a complex issue**

**Section designation:** Original Research

Yannick Dieudonné*^1,2,3^, Beatrice Uring-Lambert^4^, Mohamed Maxime Jeljeli^5,6^, Vincent Gies^7^,  Yves Alembik^8^, Anne-Sophie Korganow^1,2,3^, Aurélien Guffroy^1,2,3^.

^1^Université de Strasbourg, INSERM UMR - S1109, F-67000 Strasbourg, France

^2^Hôpitaux Universitaires de Strasbourg, Department of Clinical Immunology and Internal Medicine, National Reference Center for Systemic Autoimmune Diseases (CNR RESO), Tertiary Center for Primary Immunodeficiency, F-67000 Strasbourg, France.

^3^Université de Strasbourg, Faculty of Medicine, F-67000 Strasbourg, France.

^4^Department of Immunobiology, Hôpitaux Universitaires de Strasbourg, 67091 Strasbourg,

France.

^5^Département 3I « Infection, Immunité et Inflammation », Institut Cochin, INSERM U1016, Université de Paris, Paris, France.

^6^Université de Paris, Faculté de Médecine, AP-HP-Centre Université de Paris, Hôpital Cochin, Service d’immunologie biologique, Paris, France.

^7^Université de Strasbourg, Faculty of Pharmacy, F-67400 Illkirch, France.

^8^Department of Clinical Genetic, Hôpitaux Universitaires de Strasbourg, 67091 Strasbourg, France.

**Running Title:** Immune defect in adults with Down Syndrome

**Correspondence:**

Dr Yannick Dieudonné, MD

Université de Strasbourg, INSERM UMR - S1109, F-67000 Strasbourg, France

E-mail address: yannick.dieudonne@chru-strasbourg.fr

**SUPPLEMENTARY FIGURE LEGENDS**

**Supplemental Figure 1 |** Gating strategy used to identify peripheral B cells subsets (A), T cell subsets (B) and NK cells (C) in peripheral blood from adult patients with DS. CM, central memory; EM, effector memory; MZB, marginal zone-like B cells; NK, natural killer; P, patient; SMB, switched memory B cells; TEMRA, Terminally differentiated effector memory.

**Supplemental Table 1 | Overview of immunological abnormalities in children with DS**

|  | **Reference** |
| --- | --- |
|  |  |
| **T cell compartment** |  |
| *Thymus* |  |
| Thymic hypoplasia | [1–4] |
| Medulary expansion | [1-4] |
| Involuted Hassal’s bodies | [1-4] |
| Cystic involution | [1, 3, 4] |
| Decreased thymocytes counts | (5,6) |
| Increased double negative thymocytes frequency | [4] |
| Decreased single positive thymocytes frequency | [4, 6] |
| Increased single positive thymocytes frequency | [4] |
| Increased IFNγ secretion | (7) |
| Decreased AIRE expression | (4,8,9) |
| Decreased TRA expression | (4,9) |
| *Peripheral blood and tissues* |  |
| Decreased Naïve T cells counts | (6,10,11) |
| Increased effector/central memory T cells | (12) |
| Decreased TRECs counts | (13,14) |
| Decreased αβ T cells counts | (6,11) |
| Increased γδ T cells counts | [11] |
| Inverted CD4/CD8 ratio | (15) |
| Increased Th1/Th2 ratio | (16) |
| Increased of senescent CD57+ T cells | (17) |
| Decreased T cells IL-2 production | (18,19) |
| Decreased T cell proliferation | (20) |
| Increased T reg frequency | (4,19,21) |
| Decreased T reg suppressive fonction | [4, 21] |
| Low T cell dependant vaccinal response | (22–24) |
| **B cell compartment** |  |
| *Peripheral blood and secondary lymphoid organs* |  |
| Decreased B cell counts | (25–27) |
| Decreased naïve B cells counts | (26–28) |
| Decreased marginal zone like B cells counts | (26,29) |
| Decreased switched memory B cells counts | (27) |
| Increased CD21^low^CD38^low^counts | (26,28) |
| Increased plasmablasts counts | [26] |
| Increased BAFF serum level | [26] |
| Preserved architecture of lymphoid organs | [26] |
| Low T-cell independent vaccinal response | (3,30,31) |
| *Bone marrow* |  |
| Increased plasma cells counts | [26] |
| Decreased IgG2-IgG4 production | (18,29,32) |
| Increased IgG1-IgG3 production | (18,29,32) |
| *Peripheral tissues* |  |
| Low salivary IgA levels | (33) |
| **Innate immunity** |  |
| Physical and chemical mucosal barriers alterations | (34,35) |
| Reduced secretion drainage | (34,35) |
| Impaired ciliary function | (34,36) |
| Decreased dendritic cells counts | (37) |
| Decreased macrophages counts | (37) |
| Decreased polynuclear neutrophils counts | (36) |
| Impaired chemotaxis | (35,38–40) |
| MBL/opsonization defect | (36) |
| Decreased NK cells | (25,28) |
| Decreased iNKT cells | (36) |
|  |  |

 AIRE, autoimmune regulator; BAFF, B cell activating factor; IFN, interferon; Ig, immunoglobulin; iNKT cells, invariant natural killer T cells, MBL, mannose binding lectin; NK, natural killer; TRA, tissue restricted antigen; TREC, T cell receptor excision circle.

**Supplemental Table 2 : Characteristics of DS patients with in-depth vaccine status evaluation.**

|  | Reference values | | P1 | | P2 | | P3 | |
| --- | --- | --- | --- | --- | --- | --- | --- | --- |
|  | |  | |  | |  | |  |
| Age (years) |  | | 27 | | 19 | | 36 | |
| Sex |  | | F | | F | | M | |
| Recurrent infections before 18 years |  | | +  Bronchitis  until 8y | | +  PNA  Until 6y | | +  Bronchitis  PNA | |
| Recurrent infections after 18 years |  | | - | | - | | - | |
| Opportunistic infections |  | | - | | - | | - | |
| Autoimmune manifestations |  | | HT | | HT | | HT | |
| ANA | <1/320 | | - | | - | | 1/160 | |
| T cells (cells/µL) | 700-1,900 | | 1,086 | | 1,083 | | 514 | |
| B cells (cells/µL) | 169-271 | | 56 | | 96 | | 42 | |
| Naive B cells (cells/µL) | 112-169 | | 25 | | 81 | | 33 | |
| Transitional B cells (cells/µL) | 2-6 | | 3 | | 2 | | 1 | |
| Switched memory B cells (cells/µL) | 18-40 | | 9 | | 15 | | 3 | |
| Marginal zone-like B cells (cells/µL) | 22-54 | | 8 | | 4 | | 2 | |
| Plasmablasts (cells/µL) | 1-3 | | 1 | | 10 | | 2 | |
| IgG (g/L) | 7.2-14.7 | | 11.7 | | 13.8 | | 14.7 | |
| IgA (g/L) | 1.1-3.6 | | 3.4 | | 2.8 | | 5.0 | |
| IgM (g/L) | 0.5-3.1 | | 2.2 | | 0.8 | | 1.1 | |
| Pneumococcal vaccination status | Protective value | |  | |  | |  | |
|  |  | |  | |  | |  | |
| Time since last vaccination (years) |  | | >10 | | 4.3 | | 1.1 | |
| *S. pneumoniae* polysaccharide IgG (µg/mL) | > 1.3 | |  | |  | |  | |
| Serotype 1 |  | | 0.56 | | 0.59 | | 1.07 | |
| Serotype 3 |  | | 0.79 | | 0.38 | | 0.92 | |
| Serotype 4 |  | | 0.28 | | 1.99 | | 1.87 | |
| Serotype 5 |  | | 0.54 | | 0.82 | | 0.72 | |
| Serotype 6A |  | | 3.5 | | 0.89 | | 0.79 | |
| Serotype 6B |  | | 2.9 | | 2.4 | | 3.0 | |
| Serotype 7F |  | | 2.58 | | 2.24 | | 2.03 | |
| Serotype 9V |  | | 0.33 | | 0.32 | | 1.44 | |
| Serotype 10A |  | | 3.34 | | 13.1 | | 2.13 | |
| Serotype 12F |  | | 1.5 | | 1.33 | | 1.34 | |
| Serotype 14 |  | | 3.64 | | 1.88 | | 2.1 | |
| Serotype 15B |  | | 1.18 | | 1.73 | | 0.98 | |
| Serotype 18C |  | | 0.98 | | 1.02 | | 2.47 | |
| Serotype 19A |  | | 2.98 | | 6.31 | | 4.05 | |
| Serotype 19F |  | | 3.69 | | 5.72 | | 2.19 | |
| Serotype 23F |  | | 0.81 | | 0.56 | | 1.13 | |
| Serotypes with protective titre (n, %)* |  | | 8 (50%) | | 9 (56%) | | 11 (69%) | |
| Tetanus vaccination status | Protective value | |  | |  | |  | |
|  |  | |  | |  | |  | |
| Time since last vaccination (years) |  | | 5.7 | | 4.2 | | 6.8 | |
| Tetanus IgG (IU/mL) | > 0.15 | | 0.65 | | 0.69 | | 1.0 | |

*Immune protection was defined by an antigen-specific IgG concentration ≥1.3µg/mL for at least 70% of pneumococcal serotypes. ANA, anti-nuclear antibodies; HT, hypothyroidy. F, female; M, male; PNA, pneumonia.

**SUPPLEMENTARY REFERENCES**

1. Levin S, Schlesinger M, Handzel Z, Hahn T, Altman Y, Czernobilsky B, Boss J. Thymic deficiency in Down’s syndrome. *Pediatrics* (1979) **63**:80–87.

2. Larocca LM, Lauriola L, Ranelletti FO, Piantelli M, Maggiano N, Ricci R, Capelli A. Morphological and immunohistochemical study of Down syndrome thymus. *Am J Med Genet* (2005) **37**:225–230. doi:10.1002/ajmg.1320370745

3. Ugazio AG, Maccario R, Notarangelo LD, Burgio GR. Immunology of Down syndrome: A review. *Am J Med Genet* (2005) **37**:204–212. doi:10.1002/ajmg.1320370742

4. Marcovecchio GE, Bortolomai I, Ferrua F, Fontana E, Imberti L, Conforti E, Amodio D, Bergante S, Macchiarulo G, D’Oria V, et al. Thymic Epithelium Abnormalities in DiGeorge and Down Syndrome Patients Contribute to Dysregulation in T Cell Development. *Front Immunol* (2019) **10**: doi:10.3389/fimmu.2019.00447

5. Burgio GR, Ugazio A, Nespoli L, Maccario R. Down syndrome: a model of immunodeficiency. *Birth Defects Orig Artic Ser* (1983) **19**:325–327.

6. Murphy M, Epstein LB. Down syndrome (trisomy 21) thymuses have a decreased proportion of cells expressing high levels of TCR alpha, beta and CD3. A possible mechanism for diminished T cell function in Down syndrome. *Clin Immunol Immunopathol* (1990) **55**:453–467.

7. Murphy M, Insoft RM, Pike-Nobile L, Epstein LB. A hypothesis to explain the immune defects in Down syndrome. *Prog Clin Biol Res* (1995) **393**:147–167.

8. Lima FA, Moreira-Filho CA, Ramos PL, Brentani H, Lima L de A, Arrais M, Bento-de-Souza LC, Bento-de-Souza L, Duarte MI, Coutinho A, et al. Decreased AIRE expression and global thymic hypofunction in Down syndrome. *J Immunol Baltim Md 1950* (2011) **187**:3422–3430. doi:10.4049/jimmunol.1003053

9. Giménez-Barcons M, Casteràs A, Armengol M del P, Porta E, Correa PA, Marín A, Pujol-Borrell R, Colobran R. Autoimmune predisposition in Down syndrome may result from a partial central tolerance failure due to insufficient intrathymic expression of AIRE and peripheral antigens. *J Immunol Baltim Md 1950* (2014) **193**:3872–3879. doi:10.4049/jimmunol.1400223

10. Barrena MJ, Echaniz P, Garcia-Serrano C, Cuadrado E. Imbalance of the CD4+ Subpopulations Expressing CD45RA and CD29 Antigens in the Peripheral Blood of Adults and Children with Down Syndrome. *Scand J Immunol* (1993) **38**:323–326. doi:10.1111/j.1365-3083.1993.tb01733.x

11. Murphy M, Epstein LB. Down syndrome (DS) peripheral blood contains phenotypically mature CD3+TCR alpha, beta+ cells but abnormal proportions of TCR alpha, beta+, TCR gamma, delta+, and CD4+ CD45RA+ cells: evidence for an inefficient release of mature T cells by the DS thymus. *Clin Immunol Immunopathol* (1992) **62**:245–251.

12. Guazzarotti L, Trabattoni D, Castelletti E, Boldrighini B, Piacentini L, Duca P, Beretta S, Pacei M, Caprio C, Vigan;ago A, et al. T Lymphocyte Maturation Is Impaired in Healthy Young Individuals Carrying Trisomy 21 (Down Syndrome). *Am J Intellect Dev Disabil* (2009) **114**:100–109. doi:10.1352/2009.114.100-109

13. Prada N, Nasi M, Troiano L, Roat E, Pinti M, Nemes E, Lugli E, Ferraresi R, Ciacci L, Bertoni D, et al. Direct analysis of thymic function in children with Down’s syndrome. *Immun Ageing A* (2005) **2**:4. doi:10.1186/1742-4933-2-4

14. Bloemers BLP, Bont L, Weger RA de, Otto SA, Borghans JA, Tesselaar K. Decreased Thymic Output Accounts for Decreased Naive T Cell Numbers in Children with Down Syndrome. *J Immunol* (2011) **186**:4500–4507. doi:10.4049/jimmunol.1001700

15. Cuadrado E, Barrena MJ. Immune dysfunction in Down’s syndrome: primary immune deficiency or early senescence of the immune system? *Clin Immunol Immunopathol* (1996) **78**:209–214.

16. Franciotta D, Verri A, Zardini E, Andreoni L, De Amici M, Moratti R, Nespoli L. Interferon-gamma- and interleukin-4-producing T cells in Down’s syndrome. *Neurosci Lett* (2006) **395**:67–70. doi:10.1016/j.neulet.2005.10.048

17. Cossarizza A, Ortolani C, Forti E, Montagnani G, Paganelli R, Zannotti M, Marini M, Monti D, Franceschi C. Age-related expansion of functionally inefficient cells with markers of natural killer activity in Down’s syndrome. *Blood* (1991) **77**:1263–1270.

18. Nespoli L, Burgio GR, Ugazio AG, Maccario R. Immunological features of Down’s syndrome: a review. *J Intellect Disabil Res* (2008) **37**:543–551. doi:10.1111/j.1365-2788.1993.tb00324.x

19. Schoch J, Rohrer TR, Kaestner M, Abdul-Khaliq H, Gortner L, Sester U, Sester M, Schmidt T. Quantitative, Phenotypical, and Functional Characterization of Cellular Immunity in Children and Adolescents With Down Syndrome. *J Infect Dis* (2017) **215**:1619–1628. doi:10.1093/infdis/jix168

20. Rigas DA, Elsasser P, Hecht F. Impaired in vitro response of circulating lymphocytes to phytohemagglutinin in Down’s syndrome: dose- and time-response curves and relation to cellular immunity. *Int Arch Allergy Appl Immunol* (1970) **39**:587–608.

21. Pellegrini FP, Marinoni M, Frangione V, Tedeschi A, Gandini V, Ciglia F, Mortara L, Accolla RS, Nespoli L. Down syndrome, autoimmunity and T regulatory cells. *Clin Exp Immunol* (2012) **169**:238–243. doi:10.1111/j.1365-2249.2012.04610.x

22. Li Volti S, Mattina T, Mauro L, Bianca S, Anfuso S, Ursino A, Mollica F. Safety and effectiveness of an acellular pertussis vaccine in subjects with Down’s syndrome. *Childs Nerv Syst ChNS Off J Int Soc Pediatr Neurosurg* (1996) **12**:100–102.

23. McKay E, Hems G, Massie A, Moffat MA, Phillips KM. Serum antibody to poliovirus in patients in a mental deficiency hospital, with particular reference to Down’s syndrome. *J Hyg (Lond)* (1978) **81**:25–30. doi:10.1017/s0022172400053730

24. Philip R, Berger AC, McManus NH, Warner NH, Peacock MA, Epstein LB. Abnormalities of the in vitro cellular and humoral responses to tetanus and influenza antigens with concomitant numerical alterations in lymphocyte subsets in Down syndrome (trisomy 21). *J Immunol Baltim Md 1950* (1986) **136**:1661–1667.

25. Hingh YCM de, Vossen PW van der, Gemen EFA, Mulder AB, Hop WCJ, Brus F, Vries E de. Intrinsic Abnormalities of Lymphocyte Counts in Children with Down Syndrome. *J Pediatr* (2005) **147**:744–747. doi:10.1016/j.jpeds.2005.07.022

26. Verstegen RHJ, Driessen GJ, Bartol SJW, van Noesel CJM, Boon L, van der Burg M, van Dongen JJM, de Vries E, van Zelm MC. Defective B-cell memory in patients with Down syndrome. *J Allergy Clin Immunol* (2014) **134**:1346-1353.e9. doi:10.1016/j.jaci.2014.07.015

27. Carsetti R, Valentini D, Marcellini V, Scarsella M, Marasco E, Giustini F, Bartuli A, Villani A, Ugazio AG. Reduced numbers of switched memory B cells with high terminal differentiation potential in Down syndrome. *Eur J Immunol* (2015) **45**:903–914. doi:10.1002/eji.201445049

28. Verstegen RHJ, Kusters MAA, Gemen EFA, De Vries E. Down Syndrome B-Lymphocyte Subpopulations, Intrinsic Defect or Decreased T-Lymphocyte Help. *Pediatr Res* (2010) **67**:563–569. doi:10.1203/PDR.0b013e3181d4ecc1

29. Kusters M a. A, Verstegen RHJ, Gemen EFA, de Vries E. Intrinsic defect of the immune system in children with Down syndrome: a review. *Clin Exp Immunol* (2009) **156**:189–193. doi:10.1111/j.1365-2249.2009.03890.x

30. Costa-Carvalho BT, Martinez RMA, Dias ATN, Kubo CA, Barros-Nunes P, Leiva L, Solé D, Carneiro-Sampaio MMS, Naspitz CK, Sorensen RU. Antibody response to pneumococcal capsular polysaccharide vaccine in Down syndrome patients. *Braz J Med Biol Res Rev Bras Pesqui Medicas E Biol* (2006) **39**:1587–1592. doi:10.1590/s0100-879x2006001200010

31. Kusters M a. A, Manders NCC, de Jong B a. W, van Hout RWNM, Rijkers GT, de Vries E. Functionality of the pneumococcal antibody response in Down syndrome subjects. *Vaccine* (2013) **31**:6261–6265. doi:10.1016/j.vaccine.2013.09.070

32. Barradas C, Charlton J, MendoCa P, Lopes AI, Palha M, Trindade JC. IgG subclasses serum concentrations in a population of children with Down syndrome: comparative study with siblings and general population. *Allergol Immunopathol (Madr)* (2002) **30**:57–61.

33. Chaushu S, Chaushu G, Zigmond M, Yefenof E, Stabholz A, Shapira J, Merrick J, Bachrach G. Age-dependent deficiency in saliva and salivary antibodies secretion in Down’s syndrome. *Arch Oral Biol* (2007) **52**:1088–1096. doi:10.1016/j.archoralbio.2007.06.002

34. Verstegen RHJ, Chang KJJ, Kusters MAA. Clinical implications of immune-mediated diseases in children with Down syndrome. *Pediatr Allergy Immunol Off Publ Eur Soc Pediatr Allergy Immunol* (2019) doi:10.1111/pai.13133

35. Ram G, Chinen J. Infections and immunodeficiency in Down syndrome. *Clin Exp Immunol* (2011) **164**:9–16. doi:10.1111/j.1365-2249.2011.04335.x

36. Galati DF, Sullivan KD, Pham AT, Espinosa JM, Pearson CG. Trisomy 21 Represses Cilia Formation and Function. *Dev Cell* (2018) **46**:641-650.e6. doi:10.1016/j.devcel.2018.07.008

37. Bloemers BLP, van Bleek GM, Kimpen JLL, Bont L. Distinct abnormalities in the innate immune system of children with Down syndrome. *J Pediatr* (2010) **156**:804–809, 809.e1-809.e5. doi:10.1016/j.jpeds.2009.12.006

38. Barroeta O, Nungaray L, López-Osuna M, Armendares S, Salamanca F, Kretschmer RR. Defective monocyte chemotaxis in children with Down’s syndrome. *Pediatr Res* (1983) **17**:292–295. doi:10.1203/00006450-198304000-00013

39. Khan AJ, Evans HE, Glass L, Skin YH, Almonte D. Defective neutrophil chemotaxis in patients with Down syndrome. *J Pediatr* (1975) **87**:87–89. doi:10.1016/s0022-3476(75)80077-1

40. Novo E, García MI, Lavergne J. Nonspecific immunity in Down syndrome: a study of chemotaxis, phagocytosis, oxidative metabolism, and cell surface marker expression of polymorphonuclear cells. *Am J Med Genet* (1993) **46**:384–391. doi:10.1002/ajmg.1320460408
