# Supplementary figures and images for: Immune Defect in Adults With Down Syndrome: Insights Into a Complex Issue
Source: Front Immunol. 2020 May 8;11:840. doi: 10.3389/fimmu.2020.00840 (PMC7225335; doi:10.3389/fimmu.2020.00840)

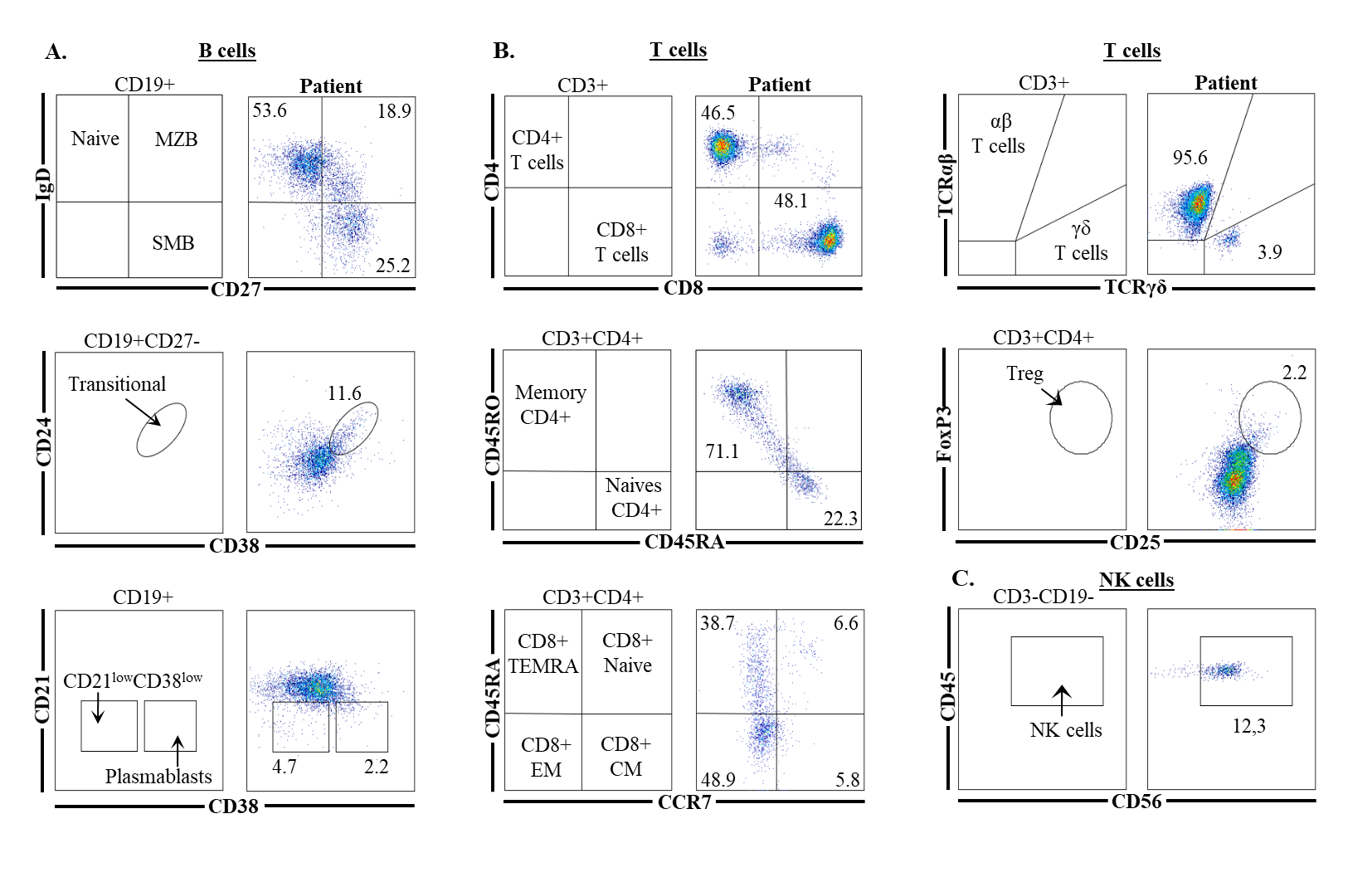

Supplement: Supplementary file 2 [file Image_1.TIF]
